# Supplementary material for: Resolving the full spectrum of human genome variation using Linked-Reads
Source: Genome Res. 2019 Apr;29(4):635–45. doi: 10.1101/gr.234443.118 (PMC6442396; doi:10.1101/gr.234443.118)
Supplement: Supplemental Material [file supp_gr.234443.118_Supplemental_longranger-2.2.zip › longranger-2.2.tar.bz2!/lr2.2-public/lib/stan/cmdstan/stan/doc/api/html/namespacemembers_func_c.html]

Stan: Namespace Members


|  |  |
| --- | --- |
|  | Stan  2.5.0  probability, sampling & optimization |


- Main Page
- Related Pages
- Namespaces
- Classes
- Files

- Namespace List
- Namespace Members

- All
- Functions
- Variables
- Typedefs
- Enumerations
- Enumerator

- a
- b
- c
- d
- e
- f
- g
- h
- i
- j
- l
- m
- n
- o
- p
- q
- r
- s
- t
- u
- v
- w


All Classes Namespaces Files Functions Variables Typedefs Enumerations Enumerator Friends Macros Pages

### - c -

- calculate\_chain()
  : stan::agrad
- categorical\_log()
  : stan::prob
- categorical\_logit\_log()
  : stan::prob
- categorical\_rng()
  : stan::prob
- cauchy\_ccdf\_log()
  : stan::prob
- cauchy\_cdf()
  : stan::prob
- cauchy\_cdf\_log()
  : stan::prob
- cauchy\_log()
  : stan::prob
- cauchy\_rng()
  : stan::prob
- cbrt()
  : stan::agrad
- ceil()
  : stan::agrad
- check\_bounded()
  : stan::math
- check\_cholesky\_factor()
  : stan::math
- check\_cholesky\_factor\_corr()
  : stan::math
- check\_column\_index()
  : stan::math
- check\_consistent\_size()
  : stan::math
- check\_consistent\_sizes()
  : stan::math
- check\_corr\_matrix()
  : stan::math
- check\_cov\_matrix()
  : stan::math
- check\_equal()
  : stan::math
- check\_finite()
  : stan::math
- check\_greater()
  : stan::math
- check\_greater\_or\_equal()
  : stan::math
- check\_ldlt\_factor()
  : stan::math
- check\_less()
  : stan::math
- check\_less\_or\_equal()
  : stan::math
- check\_lower\_triangular()
  : stan::math
- check\_matching\_dims()
  : stan::math
- check\_matching\_sizes()
  : stan::math
- check\_multiplicable()
  : stan::math
- check\_nonnegative()
  : stan::math
- check\_nonzero\_size()
  : stan::math
- check\_not\_nan()
  : stan::math
- check\_ordered()
  : stan::math
- check\_pos\_definite()
  : stan::agrad
  , stan::math
- check\_pos\_semidefinite()
  : stan::math
- check\_positive()
  : stan::math
- check\_positive\_finite()
  : stan::math
- check\_positive\_ordered()
  : stan::math
- check\_range()
  : stan::math
- check\_row\_index()
  : stan::math
- check\_simplex()
  : stan::math
- check\_size\_match()
  : stan::math
- check\_spsd\_matrix()
  : stan::math
- check\_square()
  : stan::math
- check\_std\_vector\_index()
  : stan::math
- check\_symmetric()
  : stan::math
- check\_unit\_vector()
  : stan::math
- check\_vector()
  : stan::math
- chi\_square\_ccdf\_log()
  : stan::prob
- chi\_square\_cdf()
  : stan::prob
- chi\_square\_cdf\_log()
  : stan::prob
- chi\_square\_log()
  : stan::prob
- chi\_square\_rng()
  : stan::prob
- cholesky\_corr\_constrain()
  : stan::prob
- cholesky\_corr\_free()
  : stan::prob
- cholesky\_decompose()
  : stan::math
- cholesky\_factor\_constrain()
  : stan::prob
- cholesky\_factor\_free()
  : stan::prob
- cmd\_line::bare< std::string >()
  : stan::io
- cmd\_line::val< std::string >()
  : stan::io
- col()
  : stan::math
- cols()
  : stan::math
- columns\_dot\_product()
  : stan::agrad
  , stan::math
- columns\_dot\_self()
  : stan::agrad
  , stan::math
- command()
  : stan::common
- compile()
  : stan::gm
- corr\_constrain()
  : stan::prob
- corr\_free()
  : stan::prob
- corr\_matrix\_constrain()
  : stan::prob
- corr\_matrix\_free()
  : stan::prob
- cos()
  : stan::agrad
- cosh()
  : stan::agrad
- cov\_matrix\_constrain()
  : stan::prob
- cov\_matrix\_constrain\_lkj()
  : stan::prob
- cov\_matrix\_free()
  : stan::prob
- cov\_matrix\_free\_lkj()
  : stan::prob
- crossprod()
  : stan::agrad
  , stan::math
- CubicInterp()
  : stan::optimization
- cumulative\_sum()
  : stan::math


---

[ Stan Home Page ]

*© 2011–2014,
Stan Development Team.*
